# Comparison of chloroplast genomes of *Gynura* species: sequence variation, genome rearrangement and divergence studies

**DOI:** 10.1186/s12864-019-6196-x

**Published:** 2019-10-29

**Authors:** Tianyu Han, Mimi Li, Jiawei Li, Han Lv, Bingru Ren, Jian Chen, Weilin Li

**Affiliations:** 1 Co-Innovation Center for Sustainable Forestry in Southern China, Forestry College, Nanjing Forestry University, Nanjing, 210037 China; 2 Institute of Botany, Jiangsu Province and Chinese Academy of Sciences, Nanjing, 210014 China

**Keywords:** Chloroplast genome, Genus *Gynura*, Tribe Senecioneae, Phylogenetic relationships, Divergence time

## Abstract

**Background:**

Some *Gynura* species have been reported to be natural anti-diabetic plants. Improvement of their traits towards application relies on hybridization. Clearly, phylogenetic relationships could optimize compatible hybridizations. For flowerings plants, chloroplast genomes have been used to solve many phylogenetic relationships. To date, the chloroplast genome sequences of 4 genera of the tribe Senecioneae have been uploaded to GenBank. The internal relationships within the genus *Gynura* and the relationship of the genus *Gynura* with other genera in the tribe Senecioneae need further research.

**Results:**

The chloroplast genomes of 4 *Gynura* species were sequenced, assembled and annotated. In comparison with those of 12 other Senecioneae species, the *Gynura* chloroplast genome features were analysed in detail. Subsequently, differences in the microsatellite and repeat types in the tribe were found. From the comparison, it was found that IR expansion and contraction are conserved in the genera *Gynura*, *Dendrosenecio* and *Ligularia*. Compared to other regions on the chloroplast genome, the region from 25,000 to 50,000 bp was not conserved. Seven *ndh* genes in this region are under purifying selection, with small changes in amino acids. The whole chloroplast genome sequences of 16 Senecioneae species were used to build a phylogenetic tree. Based on the oldest *Artemisia* pollen fossil, the divergence time was estimated.

**Conclusions:**

Sequencing the chloroplast genomes of 4 *Gynura* species helps us to solve many problems. The phylogenetic relationships and divergence time among 4 *Gynura* and 16 Senecioneae species were evaluated by comparing their chloroplast genomes. The phylogenetic relationship of the genera *Gynura* and *Ligularia* was different from that observed previous work. In a previous phylogenetic tree, the genus *Ligularia* belonged to the Tussilagininae subtribe, which was in a lineage that diverged earlier than other genera. Further morphology and genome-wide analyses are needed to clarify the genus relationships.

## Background

*Gynura* is a genus of flowering plants in the tribe Senecioneae of the family Asteraceae endemic to Asia, which contains 44 species in total [1]. Many species of the genus *Gynura* have been reported to have medicinal value for diabetes mellitus, such as *G. procumbens*, *G. divaricata* and *G. medica*. The aqueous extract from *G. procumbens* possesses a significant hypoglycaemic effect in streptozotocin-induced diabetic rats [2]. Additionally, an aqueous extract improved insulin sensitivity and suppressed hepatic gluconeogenesis in C57BL/KsJ-db/db mice [3]. Polysaccharide from *G. divaricata* could alleviate hyperglycaemia by modulating the activities of intestinal disaccharidases in streptozotocin-induced diabetic rats [4], and *G. divaricata*-lyophilized powder was effectively hypoglycaemic by activating insulin signalling and improving antioxidant capacity in mice with type 2 diabetes [5]. Phenolic compounds isolated from *G. medica* inhibited yeast α-glucosidase in vitro [6].

Some plants in the genus *Gynura* have also been used as vegetables and tea in people’s daily lives in East and South Asia; thus, there is value in studying the genus *Gynura*. Although *Gynura* plants are useful to resist diabetes, some shortcomings need improvement, such as the medicinal effect on diabetes, potential toxicity and oral tastes [7, 8]. Large improvement relies on interspecific hybridizations to increase genetic diversity and introgression of valuable traits. The phylogenetic relationship is useful information for interspecific hybridizations, but the phylogenetic relationship of the species in the genus *Gynura* is, as yet, unclear.

Whole chloroplast DNA ranges between 120 and 160 kb in size on the circular chromosome in most plants, composed of large single copy (LSC), small single copy (SSC), and two copies of an inverted repeat (IRa and IRb) [9, 10]. Compared to mitochondrial and nuclear genomes, chloroplast genomes are more conserved in terms of gene content, organization and structure [11]. The chloroplast genomes of angiosperms generally show slow substitution rates under adaptive evolution [12]. Considering its small size, conserved gene content and simple structure, the chloroplast genome is valid and cost-effective for studying phylogenetic relationships and the evolution of plants in different taxa. Recently, forage species of *Urochloa* [13], marine crop *Gracilaria firma* [14], epilithic sister genera *Oresitrophe* and *Mukdenia* [15], the families *Adoxaceae* and *Caprifoliaceae* of *Dipsacales* [16] were sequenced to elucidate the diversity, phylogeny and evolution of their related complete chloroplast genomes.

In the present study, we sequenced, assembled and annotated the chloroplast genomes of four *Gynura* species*.* Combined with chloroplast genomes of the genus *Dendrosenecio*, genus *Jacobaea*, genus *Ligularia* and genus *Pericallis* of the tribe Senecioneae*,* the structure features, repeat motifs, adaptive selection, phylogenetic relationships and divergence time were analysed.

## Results and discussion

### Chloroplast genome features of 16 Senecioneae species

**Fig. 1:**
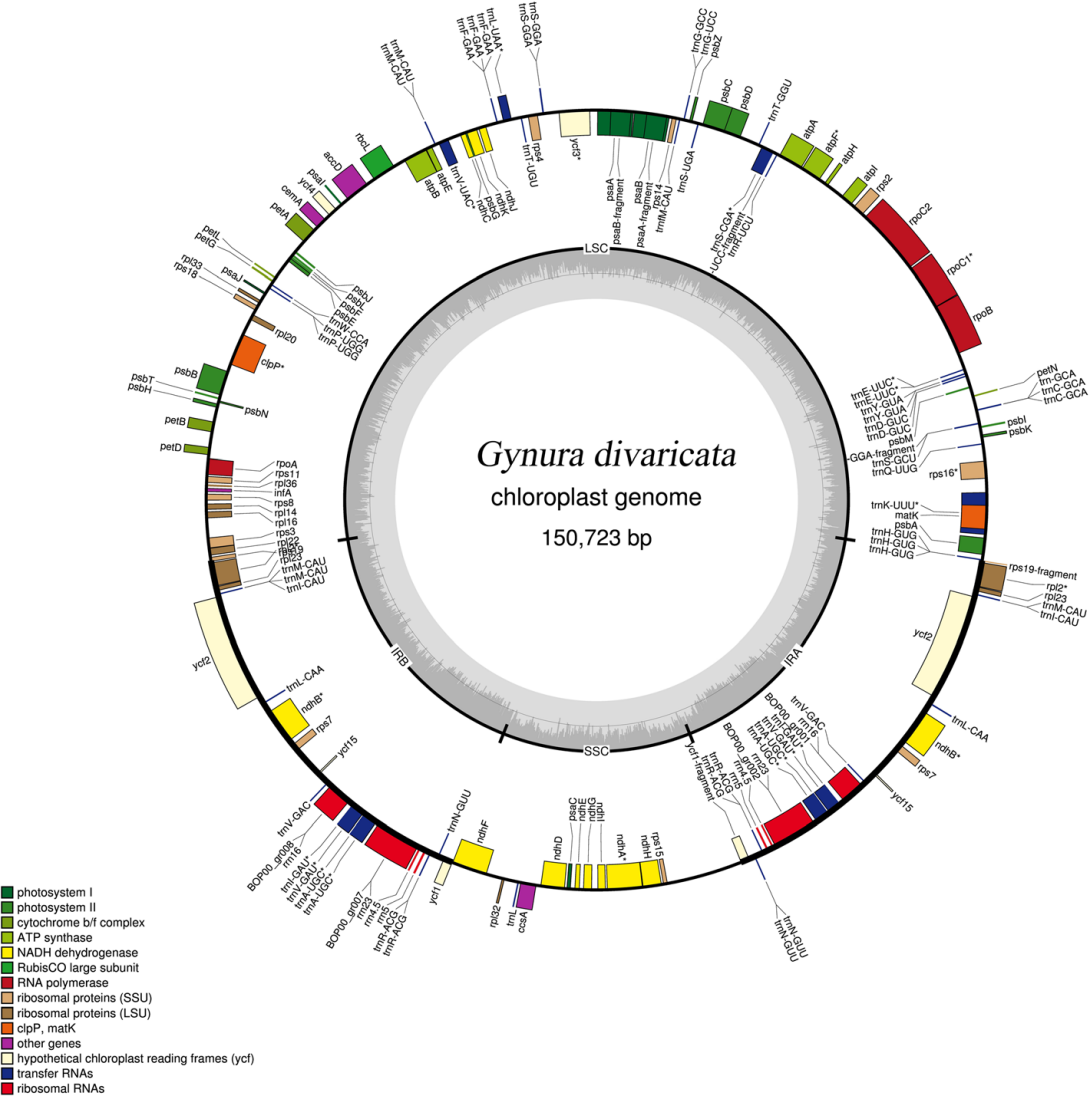
Chloroplast genome map of *Gynura divaricata.* Genes inside the circle are transcribed clockwise and genes outside are transcribed counter-clockwise. The ratio of light gray inside to drak gray outside represents the ratio of AT/CG content. The colors of different genes correspond to different functional groups in the legend

In this study, we sequenced and assembled the chloroplast genome of *Gynura bicolor*, *Gynura divaricata, Gynura formosana* and *Gynura pseudochina*. The 4 chloroplast genomes were successfully assembled and the details of data are shown in Table 1. The genus *Gynura* belongs to the tribe Senecioneae, which is the largest tribe of the family Asteraceae. Although the tribe comprises approximately 500 genera and 3000 species [17], we found that only 4 genera of the tribe Senecioneae had published chloroplast genomes in GenBank, and their IDs are listed in the methods. Five species of the genus *Dendrosenecio*, one species of the genus *Jacobaea*, five species of the genus *Ligularia*, one species of the genus *Pericallis* and four species of the genus *Gynura* were used to find their similarities and differences. The whole-sequence lengths ranged from 150,551 bp (*Dendrosenecio brassiciformis*) to 151,267 bp (*Pericallis hybrida*). With the typical quadripartite parts, such as most land plants, the chloroplast genome has one large single copy (LSC), one short single copy (SSC), and two inverted regions (IRa and IRb) (Fig. 1). The LSC lengths ranged from 82,816 bp (*Jacobaea vulgaris*) to 83,458 bp (*Dendrosenecio cheranganiensis*), the SSC lengths ranged from 17,749 bp (*D. brassiciformis*) to 18,331 bp (*P. hybrida*) and the IR lengths both ranged from 24,688 bp (*D. brassiciformis*) to 24,845 bp (*P. hybrida*) (Table 2). The total length changes were not consistent with the length changes of each region. *J. vulgaris* has the shortest chloroplast genome length, but its SSR region is longer than that of 4 *Gynura* species. In addition, there are 95 coding genes in the chloroplast genome of *P. hybrida* and 87 coding genes in *J. vulgaris.* GC content has a very low range of variation between 37.2 and 37.5%*.* Only the rRNA number is conserved in the chloroplast genome of the tribe *Senecioneae*, which is the same as that of the families *Adoxaceae* and *Caprifoliaceae* [16] but different from that of the genera *Oresitrophe* and *Mukdenia* [15].

**Table 1:** Assembling datas of 4 *Gynura* species

Speices	Raw data (Gb)	Clean data (Gb)	Total reads (bp)	Aligned reads (bp)	Assembled reads (bp)	Average coverage (Depth)
*Gynura bicolor*	8.221	8.211	10,036,414	655,986	369,592	652X
*Gynura divaricata*	7.514	7.489	10,036,414	151,500	120,390	150X
*Gynura formosana*	3.213	3.213	9,064,960	103,304	73,720	102X
*Gynura pseudochina*	3.449	3.444	9,728,392	211,624	143,102	211X

**Table 2:** Overview of chloroplast genome of 16 Senecioneae species

Speices	Size (bp)	LSC (bp)	SSC (bp)	IR (bp)	GC%	Protein	rRNA	tRNA	Total genes
*Gynura bicolor*	150,930	83,258	18,128	24,772	37.2	91	8	35	134
*Gynura divaricata*	150,723	82,998	18,163	24,781	37.2	91	8	35	134
*Gynura formosana*	151,104	83,368	18,164	24,786	37.2	91	8	35	134
*Gynura pseudochina*	151,023	83,330	18,131	24,781	37.2	91	8	35	134
*Dendrosenecio brassiciformis*	150,551	83,426	17,749	24,688	37.5	89	8	37	134
*Dendrosenecio cheranganiensis*	150,606	83,458	17,768	24,690	37.5	89	8	37	134
*Dendrosenecio johnstonii*	150,607	83,471	17,756	24,690	37.4	89	8	37	134
*Dendrosenecio kilimanjari*	150,593	83,457	17,756	24,690	37.5	89	8	37	134
*Dendrosenecio meruensis*	150,587	83,450	17,757	24,690	37.5	89	8	37	134
*Jacobaea vulgaris*	150,689	82,816	18,277	24,798	37.3	87	8	37	132
*Ligularia hodgsonii*	151,136	83,254	18,218	24,832	37.5	94	8	36	138
*Ligularia intermedia*	151,152	83,259	18,233	24,830	37.5	94	8	36	138
*Ligularia jaluensis*	151,148	83,264	18,226	24,829	37.5	94	8	36	138
*Ligularia mongolica*	151,118	83,245	18,215	24,829	37.5	93	8	36	137
*Ligularia veitchiana*	151,253	83,331	18,248	24,837	37.5	94	8	36	138
*Pericallis hybrida*	151,267	83,246	18,331	24,845	37.3	95	8	36	139

### Microsatellite and repeat types

**Fig. 2:**
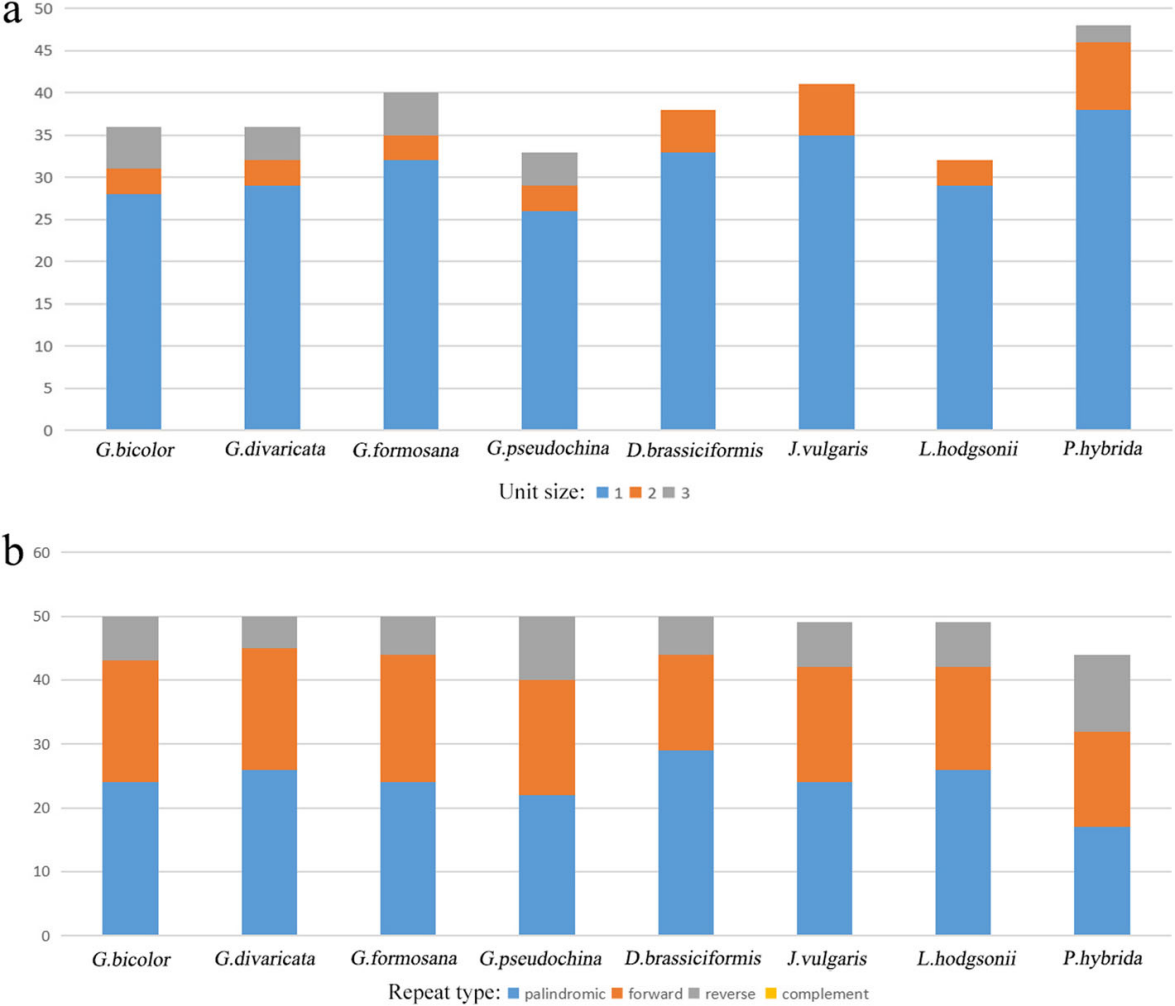
The repeat motif statistics of 7 Senecioneae species*.* **a** Frequency of repeat types. **b** Frequency of unit size

The number of microsatellites with mono-, di- and trinucleotide repeat motifs varies in the tribe. *D. brassiciformis*, *J. vulgaris* and *L. hodgsonii* do not have trinucleotide repeat motifs, while four *Gynura* species have 4 to 5 trinucleotide repeat motifs. The number of mononucleotide repeat motifs is 28 to 38, accounting for the largest proportion (Fig. 2a). The unit size of microsatellites is significantly different in four *Urochloa* species [13], which have tetranucleotide repeat motifs, and the trinucleotide motif is the largest proportion. The total number of repeat types is consistent with that in the four *Gynura* species, but the number of each repeat type is different. Palindromic repeats are the most abundant, and complement repeats are secondary in 16 Senecioneae species (Fig. 2b). Comparing the *Oresitrophe* and Senecioneae species [15], the Senecioneae species have 5 to 12 reverse repeats, but the *Oresitrophe* species do not have reverse repeats. In addition, the forward and palindromic repeat numbers are similar in the *Oresitrophe* species.

### Contraction and expansion of inverted repeats

**Fig. 3:**
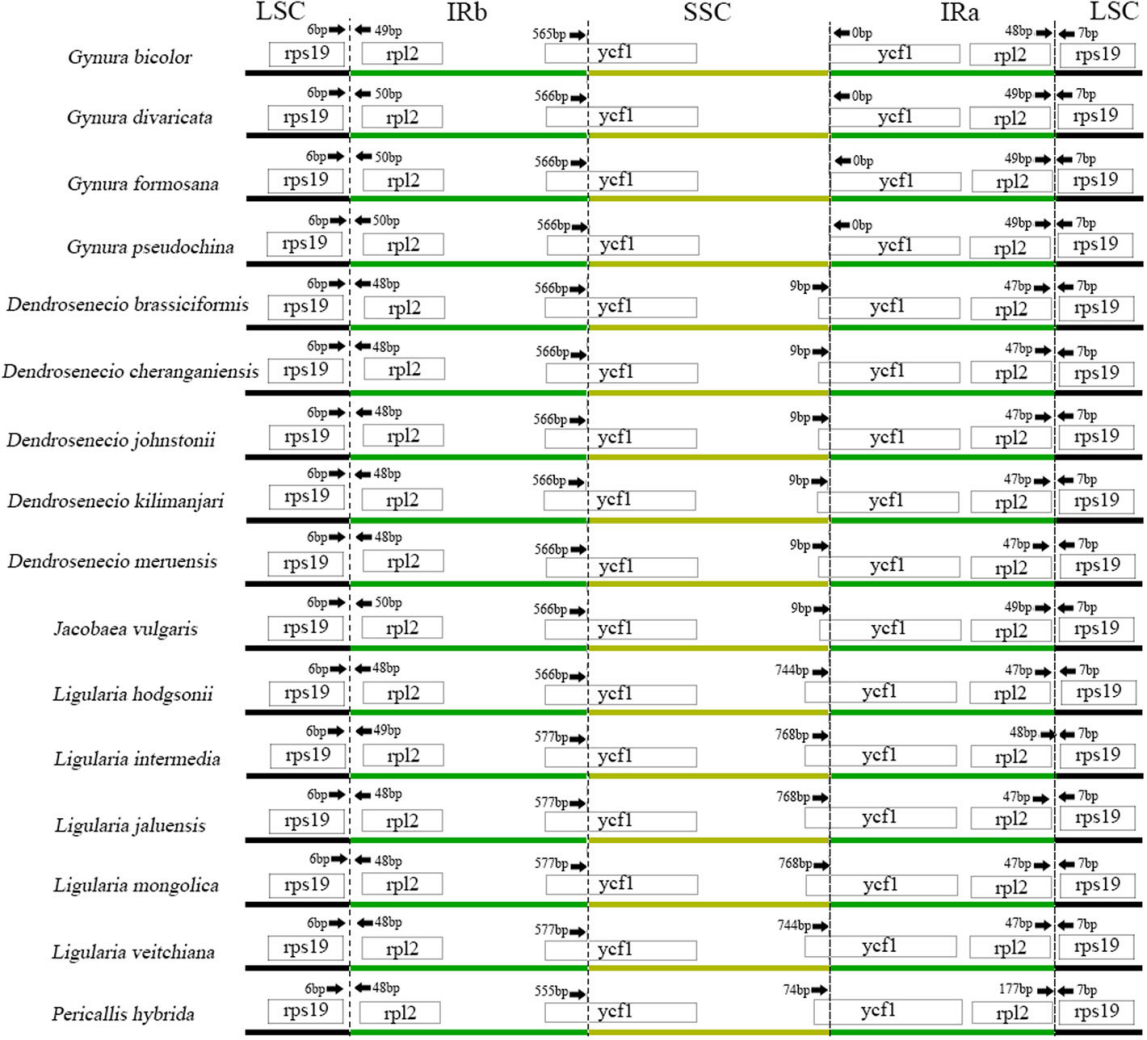
Schematic representation of the border positions of LSC, IRs and SSC in the chloroplast genome of 16 Senecioneae species

The chloroplast genome is highly conserved in land plants, but IR expansion and contraction lead to different genome sizes in different plants [18]. The LSC/IRb/SSC/IRa/LSC border and adjacent genes of 16 species of the Senecioneae tribe were carefully analysed to find similarities and differences (Fig. 3). The *rps19* and *rpl2* genes are located in the LSC/IRb and IRa/LSC borders in pairs. In 16 Senecioneae species, the two copies of *rps19* have no change in position in relation to the border, and the two copies of *rpl2* are relatively conserved, with 1–3 base position changes except for the IRa/LSC border of *P. hybrida*. One copy of *ycf1* spans the border of LSC/IRb, and another copy is different. The start position is just on the border in the four *Gynura* species, but the others also span the border of IRa/LSC. By comparison, IR expansion and contraction are conserved in the genera *Gynura*, *Dendrosenecio* and *Ligularia*.

### Sequence variation and adaptive selection

**Fig. 4:**
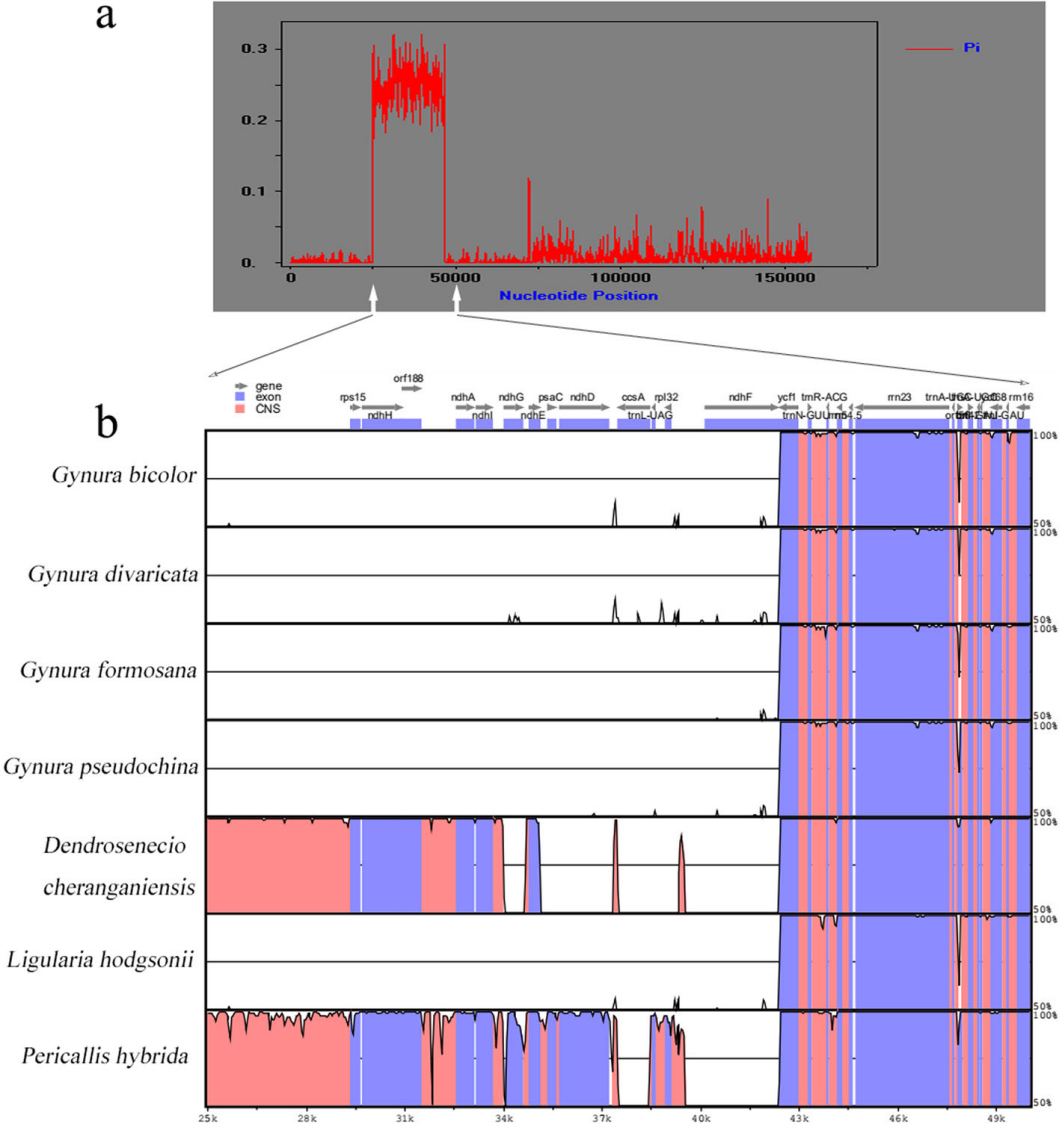
Sequence divergence of chloroplast genome sequences in 7 Senecioneae species*.* **a** The Pi value (nucleotide diversity) of the 7 chloroplast genome sequences. **b** The sequence divergence from 25,000 bp to 50,000 bp visualized by mVISTA program. The vertical scale indicates percentage identity, ranging from 50 to 100%

The whole chloroplast genome sequences of 16 Senecioneae species were aligned by the MAFFT program to find sequence variation. The alignment result was used to calculate the DNA polymorphism by the DnaSP program. The base sequence has a Pi value (nucleotide diversity) of 0.2–0.3 at 25,000 bp to 50,000 bp, and that of other positions was below 0.1 (Fig. 4a). This result shows that this region is not conserved, similar to other regions of the chloroplast genome. For further analysis of the results, the chloroplast genome sequences of four *Gynura* species, *D. cheranganiensis*, *L. hodgsonii* and *P. hybrida* were aligned and visualized by the mVISTA program. The overall result is consistent with the DNA polymorphism result (Additional file 1: Figure S1) and shows that the region from 25,000 bp to 50,000 bp is not conserved, similar to other regions. The four *Gynura* species are conserved in the 25,000 bp to 50,000 bp region and are similar to *L. hodgsonii.* In that region, *D. cheranganiensis* is close to *P. hybrida* but different from the four *Gynura* species and *L. hodgsonii* (Fig. 4b)*.* That region has a total of 12 genes, and 7 genes encode the NAD(P) H dehydrogenase (NDH) complex subunit. The function of the NAD(P) H dehydrogenase (NDH) complex is well known in photosystem I (PSI) cyclic electron flow (CEF) and chlororespiration [19, 20], so the substitution of *ndh* genes was further studied. The ratio of the non-synonymous (dN)/synonymous substitution (dS) rate was calculated by the PAML program. A ratio > 1 indicates positive selection, a ratio < 1 indicates purifying selection and a ratio = 1 indicates neutral evolution. All the dN/dS ratios of 7 genes below 1 indicate that they are under purifying selection, and little amino acid change occurred (Table 3). Thus, the functions of 7 *ndh* genes should be conserved during evolution, although they are not located in a conserved region.

**Table 3:** Molecular evolutionary rate of 7 *ndh* genes in 16 Senecioneae species

Gene	*ndhA*	*ndhB*	*ndhC*	*ndhD*	*ndhE*	*ndhF*	*ndhG*	*ndhH*	*ndhI*	*ndhJ*	*ndhK*
dN/dS	0.05971	0.71728	0.16003	0.34997	0.0001	0.33121	0.39231	0.13607	0.0001	0.06077	0.3433

### Phylogenetic relationships

**Fig. 5:**
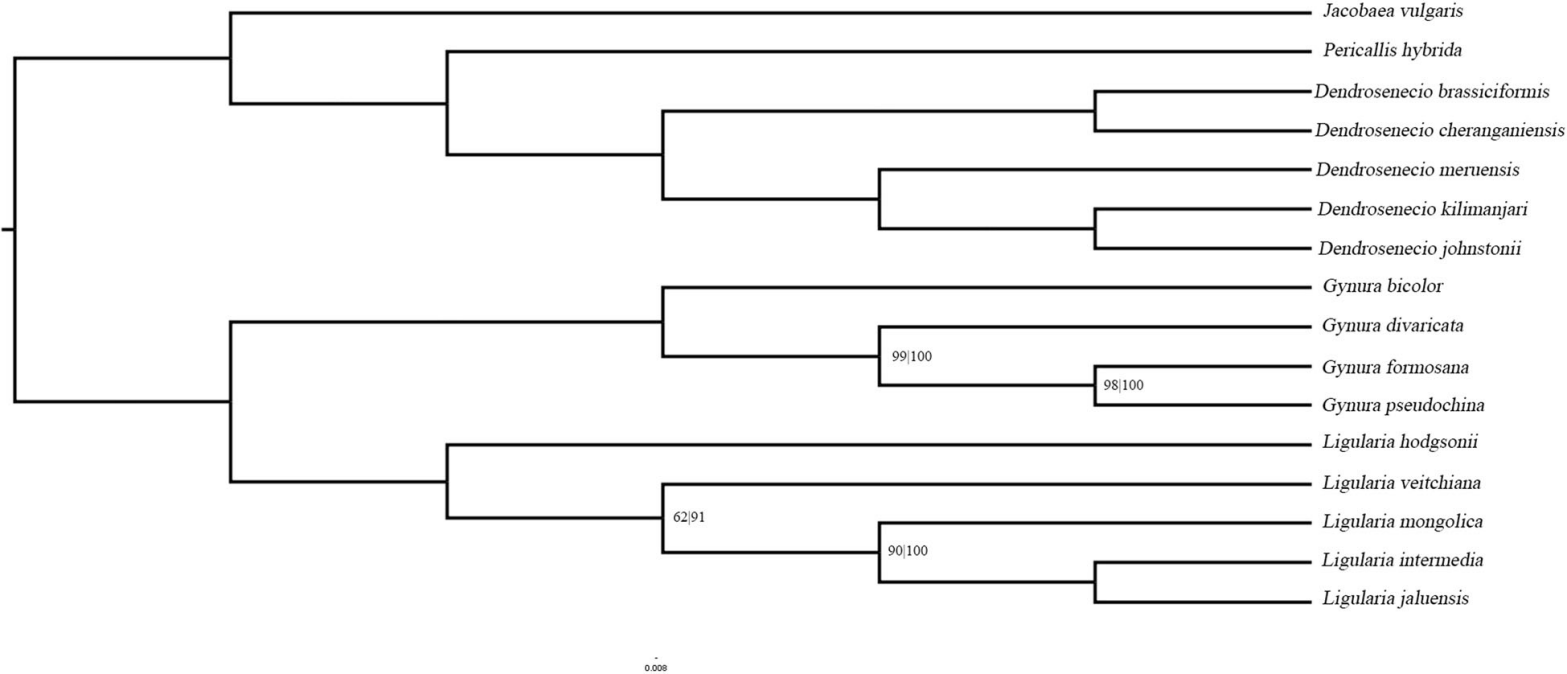
Maximum-likelihood (ML) phylogenetic tree obtained for 16 Senecioneae species based on the whole chloroplast genome sequences. Unlabeled nodes have bootsrap values of 100%. Noted nodes shows the bootstrap values of ML/BI

A sequence alignment of 16 Senecioneae species was used to construct a maximum likelihood (Fig. 5) and Bayesian inference (Additional file 2: Figure S2) tree. In the ML tree, two major clades were constructed with a 100% bootstrap value. One clade includes the genera *Gynura* and *Ligularia*, and the other clade includes the genera *Dendrosenecio*, *Pericallis* and *Jacobaea.* In the genus *Gynura, G. bicolor* was the first to differentiate, followed by *G. divaricata* and, finally, *G. formosana* and *G. pseudochina*. The former systemic phylogenies of the tribe Senecioneae based on the ITS region (nuclear) and plastid fragment sequences show a significant difference from the phylogenetic tree [17]. In a previous phylogenetic tree, the genus *Ligularia* belongs to the Tussilagininae subtribe, which was in lineage that diverged earlier than other genera. The sequence is relatively conserved among four *Gynura* species and five *Ligularia* species, and the Pi value of most sequence locations is below 0.1 (Additional file 3: Figure S3), which is significantly lower than that of the 16 species alignment. From the perspective of whole chloroplast genomes, the genus *Ligularia* is close to the genus *Gynura*.

### Divergence time estimation

**Fig. 6:**
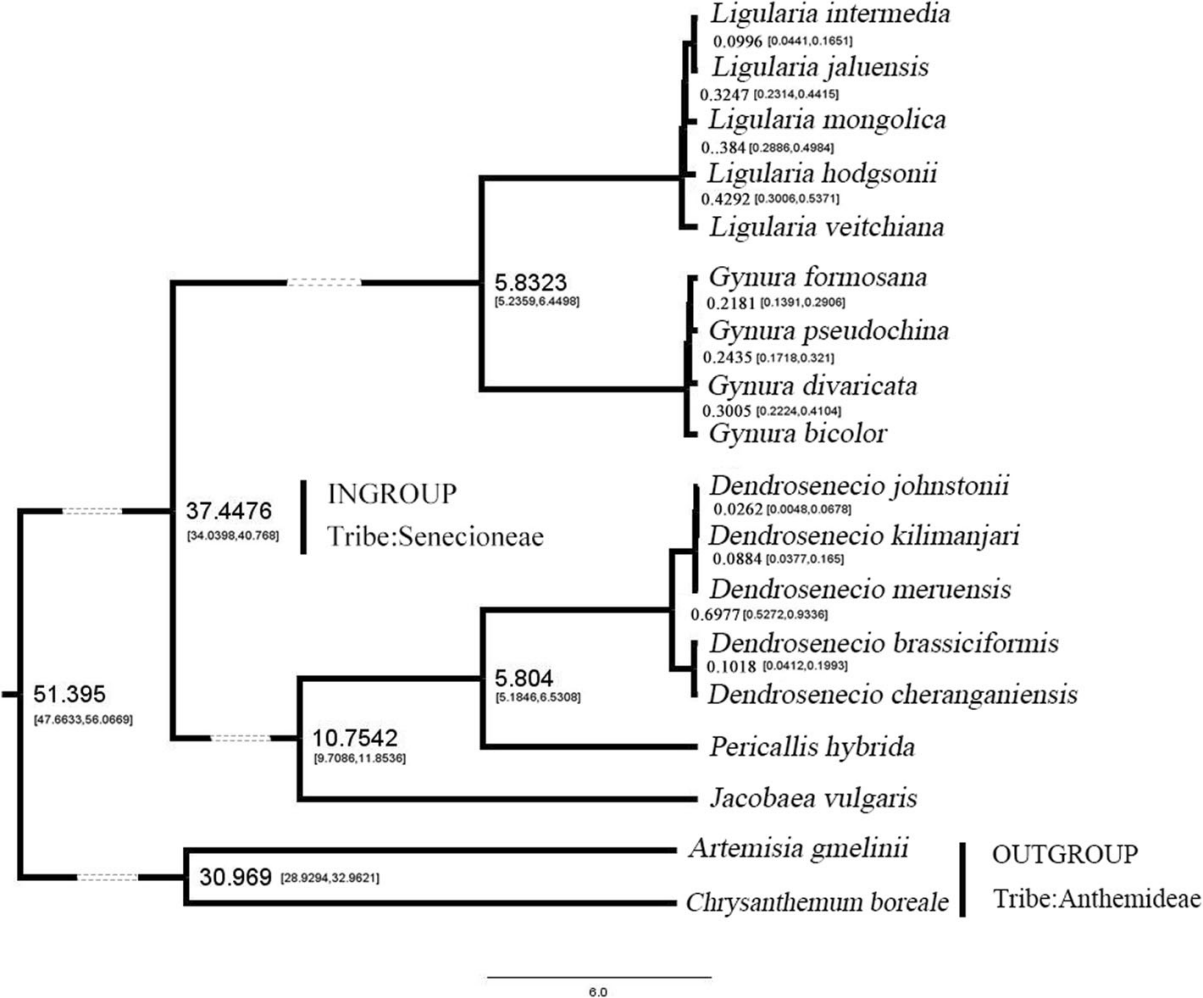
Divergence time estimation of 18 *Asteraceae* species. Dotted lines shorten the proportional length. The left and right numbers in square brackets are 95% HPD upper and lower bound respectively

For the divergence time estimation of the 16 Senecioneae species, *Artemisia gmelinii* and *Chrysanthemum boreale* (tribe Anthemideae) were selected as the outgroup due to the oldest *Artemisia* fossil pollen [21, 22]. The divergence time of 16 Senecioneae species was estimated by the BEAST2.0 program (Fig. 6). The divergence clades of these genera are the same as the ML tree. The two major clades were expected to differentiate 37.4 mya (late Eocene). Both *Gynura* and *Ligularia* differentiated 5.8 mya (late Miocene). *Dendrosenecio* and *Pericallis* also differentiated 5.8 mya. The divergence time of the tribes *Senecioneae* and *Anthemideae* was 51.39 mya (early Eocene), and the result was consistent with that of a previous study on the evolution and phylogenetic of the family *Asteroideae* based on plastid fragment sequences [22]. The traditional view on divergence time of the genus *Gynura* is in the Old World after the Atlantic opening. In that time, the senecioid species were transferred to South America, and divergence began [23]. The divergence time of *Gynura* species was approximately 0.3 mya, and the result showed that the divergence time of the genus *Gynura* was much earlier than that of the traditional view. The divergence time of the genus *Gynura* could not start at hundreds or thousands of years ago [23], and the divergence time estimated by the BEAST program was in the same time period as that of other genera of land plants [13–16].

## Conclusion

This study analyses the chloroplast genome of four *Gynura* species used as herbal medicine in parts of Asia. By comparing with other plants in the tribe Senecioneae, the repeat motifs, detailed structure features, phylogenetic relationships and divergence time estimation were investigated. The phylogenetic relationships of the genera *Gynura* and *Ligularia* and others are still in doubt. The tribe Senecioneae contains 155 genera and is almost distributed throughout the world [17]. The phylogenetic relationship is difficult to determine by chloroplast fragment or genome. Morphology and genome-wide analyses are needed to further clarify the genus relationships. Determining interspecific relationships and intergeneric relationships will facilitate hybrid breeding of *Gynura* species.

## Methods

### Plant materials, genome sequencing and assembly

*Gynura bicolor*, a cultivated plant, voucher specimen (510918–1), was collected from Nanjing Botanical Garden Mem. Sun Yet-Sen. *Gynura divaricata*, a cultivated plant, voucher specimen (510918–6), was collected from Nanjing Botanical Garden Mem. Sun Yet-Sen. *Gynura formosana*, a cultivated plant, voucher specimen (512019–3), was collected from Kunming Botanical Garden. *Gynura pseudochina,* a wild plant, voucher specimen (512019–8), was collected from Wenshan Zhuang and Miao Autonomous Prefecture, Yunnan Province. All the plants were collected by Prof. Bingru Ren, and the specimens were deposited in the Herbarium of Institute of Botany, Jiangsu Province and Chinese Academy of Sciences.

The *Gynura bicolor*, *G. divaricata*, *G. formosana* and *G. pseudochina* plants were grown in a greenhouse with normal sunlight and temperature. The DNA was extracted from their fresh leaves by the CTAB method [24], and DNA degradation and contamination were monitored on 1% agarose gels.

Approximately 1.5 μg of the DNA sample was fragmented by sonication to a size of 350 bp. Then, the DNA fragments were end polished, poly A-tailed, and ligated with a full-length adaptor for Illumina sequencing, with further PCR amplification. After PCR product purification (AMPure XP system), libraries were analysed for size distribution by an Agilent 2100 Bioanalyzer and quantified by using real-time PCR.

The libraries constructed above were sequenced by the Illumina HiSeq X Ten platform, and 150 bp paired-end reads (PE150) were generated with an insert size of approximately 350 bp. Quality control (QC) removed reads with ≥10% unidentified nucleotides (N), > 50% bases having a phred quality < 5 and > 10 nt aligned to the adaptor, allowing ≤10% mismatches.

The Perl script NOVOPlasty 2.7.2 [25] was used to assemble the chloroplast genome sequence with a 50 K-mer. The chloroplast genome sequence of *Dendrosenecio cheranganiensis* (tribe Senecioneae) was selected as the reference genome. The family Asteraceae plant sequences used in the study were downloaded from GenBank as follows: *Dendrosenecio brassiciformis* (NC_037960.1), *Dendrosenecio cheranganiensis* (NC_037956.1), *Dendrosenecio johnstonii* (NC_037959.1), *Dendrosenecio kilimanjari* (NC_037957.1), *Dendrosenecio meruensis* (NC_037958.1), *Jacobaea vulgaris* (NC_037957.1), *Ligularia hodgsonii* (NC_039381.1), *Ligularia intermedia* (NC_039382.1), *Ligularia jaluensis* (NC_039383.1), *Ligularia mongolica* (NC_039384.1), *Ligularia veitchiana* (NC_039385.1), *Artemisia gmelinii* (NC_031399.1), and *Chrysanthemum boreale* (NC_037388.1).

### Chloroplast genome annotation

The whole chloroplast genome sequences were annotated by Dual Organellar Genome Annotator [26] and GeSeq [27] with default parameters. Chloroplast genome sequences of tribe *Senecioneae* plants *Dendrosenecio cheranganiensis* and *Pericallis hybrida* were used as reference sequences. Subsequently, all tRNAs were verified by ARAGORN v1.2.38 [28] and tRNAscan-SE v2.0 [29]. A schematic diagram of the chloroplast genome with annotations was obtained by OGDRAW [30].

### Repeat structure analysis

The microsatellite regions are a tract of repetitive DNA in which certain DNA motifs (ranging in length from 1 to 6 or more base pairs) are repeated, typically 5–50 times [31, 32]. The Perl script Microsatellite identification tool (MISA, http://pgrc.ipk-gatersleben.de/misa/misa.html) was used to find the microsatellite regions of the chloroplast genome. Considering the features of plant chloroplasts, the numbers of each unit of continuous DNA motifs was set to 1–6, and the minus DNA motifs of each unit was 1–10, 2–6, 3–5, 4–5, 5–5, and 6–5. Forward, reverse, complement and palindromic repeat types were detected by the online tool REPuter [33]. The Hamming distance was set as 1, and the minimum repeat size was 30 bp.

### Chloroplast genome analysis

All the chloroplast genome sequences were aligned by MAFFT7.427 [34] on the FFT-NS-2 module. The different chloroplast genome sequences (LSC, SSC, IRa and IRb) concatenated together to make one sequence per species. Alignments of 7 selected genome sequences were visualized by mVISTA [35]. DNA polymorphism (nucleotide diversity) was calculated by DnaSPv5 [36] based on alignment results.

Molecular evolutionary rates (ω) between orthologous genes were estimated by calculating the ratio of the non-synonymous (dN)/synonymous substitution (dS) rates. Coding gene sequences of selected regions were extracted by using Artemis [37]. Gene sequences of each species were aligned by Clustal X [38] with default parameters, and the alignment results (dnd format) were converted to PML format by DAMBE [39] for subsequent analysis. The dN/dS value was calculated by the codeml module (seqtype = 1, model = 0, Nsites = 1,7,8) in PAML4.9i [40]. Significant differences were calculated by the likelihood ratio test.

### Phylogenetic analysis

The 16 chloroplast genome sequences of the tribe Senecioneae (family Asteraceae) were aligned by MAFFT, and the results were used to analyse the phylogenetic relationships. RAxML8 [41] was used to build a maximum likelihood tree with the GTRGAMMAI module and 1000 bootstrap replicates. Mrbayes3.2.7a [42] was used to build a Bayesian inference tree. The parameter settings were as follows: nst = 6, rates = invgamma, burnin = 500, Ngen = 20,000, Samplefreq = 10, and Printfreq = 100. Both the results of the ML tree and BI tree were visualized by FigTree V1.4.3 (http://tree.bio.ed.ac.uk/software/figtree/).

### Divergence time estimation

The divergence time of 16 species was estimated by BEAST2 [43]. The oldest *Artemisia* fossil pollen has been recorded from the Eocene–Oligocene boundary [21, 44]. The Asteraceae family plants *Artemisia gmelinii* and *Chrysanthemum boreale* were selected as the outgroup, and the node *Artemisia*–*Chrysanthemum* was constrained by using a lognormal distribution with an offset of 31 Ma and a mean and standard deviation of 0.5 [22]. The HKY nucleotide substitution model and the prior tree Yule model were selected with a strict clock. Each MCMC run had a chain length of 100,000,000 with sampling every 10,000 steps. Tracer [45] was used to read the ESS and trace value of logged statistics to access the results. Then, the divergence time was accessed by the Treeannotator program of BEAST2. The detailed settings were as follows: burnin percentage = 50, posterior probability limit = 0.0, target tree type = maximum clade credibility tree, and node heights = mean heights.

## Supplementary information

**Additional file 1: Figure S1.** Alignment of whole choloroplast genome sequences of 7 Senecioneae species. The vertical scale indicates percentage identity, ranging from 50 to 100%.

**Additional file 2: Figure S2.** Bayesian inference (BI) phylogenetic tree obtained for 16 Senecioneae species based on the whole chloroplast genome sequences. Unlabeled nodes have bootsrap values of 100%.

**Additional file 3: Figure S3.** The Pi value (nucleotide diversity) of chloroplast genome sequences between four *Gynura* species and five *Ligularia* species.

## Data Availability

All the data and materials are available from the corresponding authors upon request. The raw sequence data was uploaded to NCBI SRA database and the unique identifier is BioProject: PRJNA577235.
