# Supplementary figures and images for: Comparison of chloroplast genomes of Gynura species: sequence variation, genome rearrangement and divergence studies
Source: BMC Genomics. 2019 Oct 29;20:791. doi: 10.1186/s12864-019-6196-x (PMC6821010; doi:10.1186/s12864-019-6196-x)

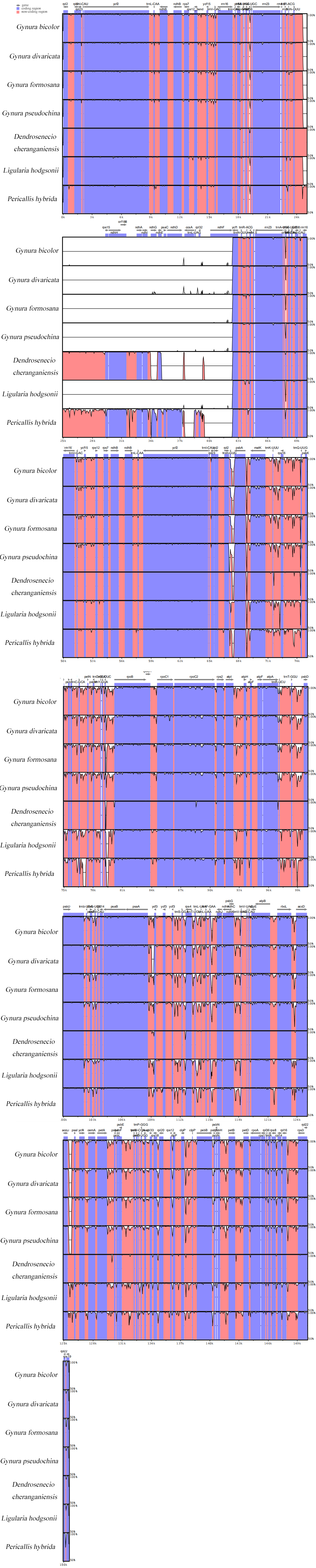

Supplement: Supplementary file 1 — Additional file 1: Figure S1. Alignment of whole choloroplast genome sequences of 7 Senecioneae species. The vertical scale indicates percentage identity, ranging from 50 to 100%. [file 12864_2019_6196_MOESM1_ESM.jpg]

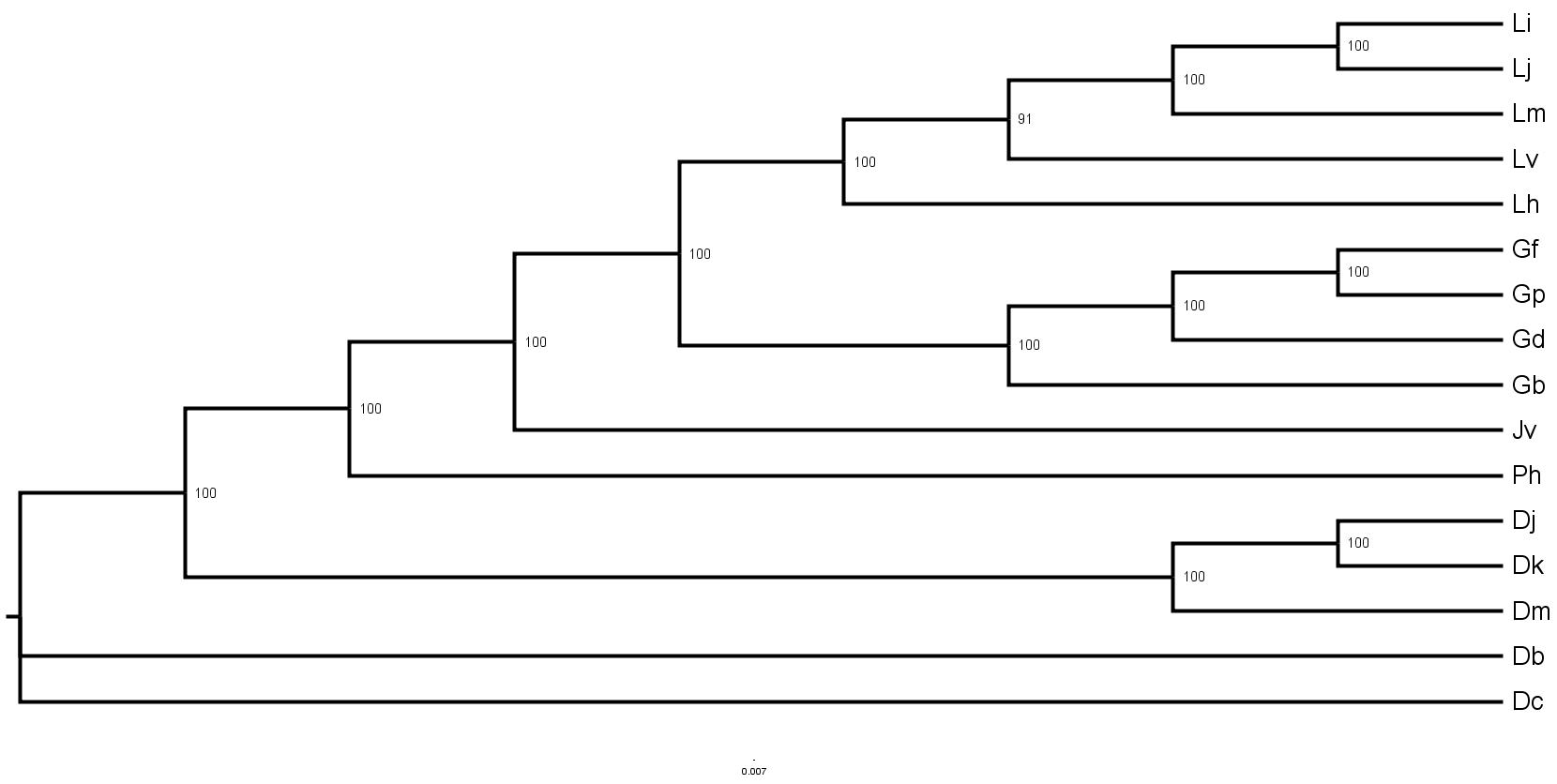

Supplement: Supplementary file 2 — Additional file 2: Figure S2. Bayesian inference (BI) phylogenetic tree obtained for 16 Senecioneae species based on the whole chloroplast genome sequences. Unlabeled nodes have bootsrap values of 100%. [file 12864_2019_6196_MOESM2_ESM.jpg]

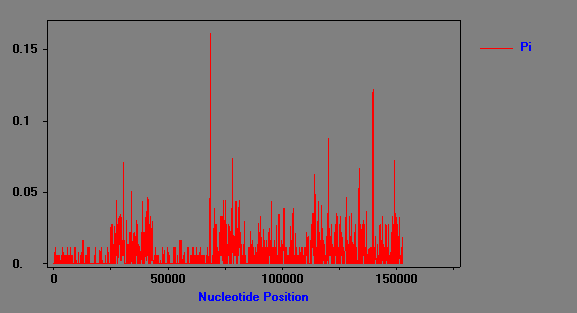

Supplement: Supplementary file 3 — Additional file 3: Figure S3. The Pi value (nucleotide diversity) of chloroplast genome sequences between four Gynura species and five Ligularia species. [file 12864_2019_6196_MOESM3_ESM.bmp]
